# Supplementary material for: Exploring a combined biomarker for tuberculosis treatment response: protocol for a prospective observational cohort study
Source: BMJ Open. 2021 Jul 8;11(7):e052885. doi: 10.1136/bmjopen-2021-052885 (PMC8268918; doi:10.1136/bmjopen-2021-052885)
Supplement: Author's manuscript [file bmjopen-2021-052885.draft_revisions.pdf]

BMJ Open

BMJ Open is committed to open peer review. As part of this commitment we make the peer review history of every article we publish publicly available.

When an article is published we post the peer reviewers' comments and the authors' responses online. We also post the versions of the paper that were used during peer review. These are the versions that the peer review comments apply to.

The versions of the paper that follow are the versions that were submitted during the peer review process. They are not the versions of record or the final published versions. They should not be cited or distributed as the published version of this manuscript.

BMJ Open is an open access journal and the full, final, typeset and author-corrected version of record of the manuscript is available on our site with no access controls, subscription charges or pay-per-view fees (http://bmjopen.bmj.com).

If you have any questions on BMJ Open's open peer review process please email info.bmjopen@bmj.com

BMJ Open

Exploring a combined bio-marker for tuberculosis treatment response; a prospective observational cohort study protocol

Journal:	*BMJ Open*
Manuscript ID	bmjopen-2021-052885
Article Type:	Protocol
Date Submitted by the Author:	28-Apr-2021
Complete List of Authors:	Kloprogge, Frank; University College London<br>Abubakar, Ibrahim; Health Protection Agency, Tuberculosis Section, Centre for Infections<br>Esmail, Hanif; University College London,<br>Hack, Vanessa ; University College London, Institute for Global Health<br>Kunst, Heinke; Queen Mary University of London, Department of Respiratory Medicine<br>McHugh, Timothy; Centre for Clinical Microbiology, Research Department of Infection, Royal Free Campus, University College London<br>Noursadeghi, Mahdad; University College London<br>Surey, Julian; University College London<br>Tiberi, Simon; Barts Health NHS Trust, Blizard Institute, Queen Mary University of London, Department of Infection<br>Lipman, Marc; Royal Free Campus, Respiratory Medicine
Keywords:	Tuberculosis < INFECTIOUS DISEASES, BACTERIOLOGY, CLINICAL PHARMACOLOGY

SCHOLARONE™
Manuscripts

I, the Submitting Author has the right to grant and does grant on behalf of all authors of the Work (as defined in the below author licence), an exclusive licence and/or a non-exclusive licence for contributions from authors who are: i) UK Crown employees; ii) where BMJ has agreed a CC-BY licence shall apply, and/or iii) in accordance with the terms applicable for US Federal Government officers or employees acting as part of their official duties; on a worldwide, perpetual, irrevocable, royalty-free basis to BMJ Publishing Group Ltd ("BMJ") its licensees and where the relevant Journal is co-owned by BMJ to the co-owners of the Journal, to publish the Work in this journal and any other BMJ products and to exploit all rights, as set out in our licence.

The Submitting Author accepts and understands that any supply made under these terms is made by BMJ to the Submitting Author unless you are acting as an employee on behalf of your employer or a postgraduate student of an affiliated institution which is paying any applicable article publishing charge ("APC") for Open Access articles. Where the Submitting Author wishes to make the Work available on an Open Access basis (and intends to pay the relevant APC), the terms of reuse of such Open Access shall be governed by a Creative Commons licence – details of these licences and which Creative Commons licence will apply to this Work are set out in our licence referred to above.

Other than as permitted in any relevant BMJ Author's Self Archiving Policies, I confirm this Work has not been accepted for publication elsewhere, is not being considered for publication elsewhere and does not duplicate material already published. I confirm all authors consent to publication of this Work and authorise the granting of this licence.

For peer review only

Exploring a combined bio-marker for tuberculosis treatment response; a prospective observational cohort study protocol

Frank Kloprogge (1), Ibrahim Abubakar (1), Hanif Esmail (1,2), Vanessa Hack (1), Heinke Kunst (3), Timothy D. McHugh (4), Mahdad Noursadeghi (5), Julian Surey (1), Simon Tiberi (3,6), Marc Lipman (7,8)

1 Institute for Global Health, University College London, United Kingdom.

2 Medical Research Council Clinical Trials Unit, University College London, London, United Kingdom.

3 Blizard Institute, Barts and The London School of Medicine and Dentistry, Queen Mary University of London, UK

4 UCL Centre for Clinical Microbiology, Division of Infection & Immunity, University College London, London, United Kingdom.

5 Division of Infection and Immunity, University College London, London, United Kingdom.

6 Division of Infection, Royal London Hospital, Barts Health NHS Trust, London, United Kingdom.

7 Royal Free London NHS Foundation Trust, London, United Kingdom.

8 UCL Respiratory, Division of Medicine, University College London, London, United Kingdom.

Corresponding author:

Frank Kloprogge

30 Guilford Street

London

WC1N 1EH

United Kingdom

Tel: +44 (0)20 7905 2889

Email: f.kloprogge@ucl.ac.uk

For peer review only

Abstract

Introduction

An improved understanding of underlying factors explaining tuberculosis (TB) treatment response is urgently needed to help clinicians optimise and personalise treatment and assist scientists undertaking novel treatment regimen trials. Promising outcome proxy measures, including sputum bacillary load and host immune response, are widely reported with variable results. However, they have not been studied together in combination with antibiotic exposure. The aim of this observational cohort study is to investigate which antibiotic exposures correlate with sputum bacillary load and which with the host immune response. Subsequently, we will explore if these correlations can be used to inform a candidate combined biomarker predicting cure.

Methods and analysis

All patients aged 18 years or above, diagnosed with drug sensitive pulmonary TB (culture or molecular test), eligible for standard anti-tuberculosis treatment, at selected London, UK TB Services will be invited to participate in this observational cohort study. Patients will be asked to give blood for host transcriptomic analysis and antibiotic plasma exposure quantification, in addition to standard of care sputum samples for bacillary load. Antibiotic plasma concentrations will be quantified using a validated LC-MS/MS assay, and sputum bacillary load by Mycobacterial Growth Incubator Tube (MGIT) time to positivity (TTP). Expression from a total of 35 prespecified host blood genes will be quantified using NanoString®. Antibiotic exposure, sputum bacillary load and host blood transcriptomic time series data will be analysed using nonlinear mixed-effects models. Correlations between combinations of longitudinal biomarkers and microbiological cure at end of treatment and remaining relapse-free for one year thereafter will be analysed using logistic regression or Cox proportional hazard models.

For peer review only

Ethics and dissemination

The observational cohort study has been approved by the UK's HRA REC. Written informed consent will be obtained. Results will be disseminated via publication, presentation and through engagement with institutes/companies developing novel anti-TB treatment combinations.

For peer review only

Article summary

1. This feasibility study explores for the first time correlations between antibiotic exposure and both sputum tuberculosis (TB) bacillary load and host immune response.
2. This enables correlations between different combinations of longitudinal markers of TB treatment response and TB treatment outcome to be explored.
3. Such correlations can form the basis for a candidate biomarker that predicts cure from TB during the intensive phase of treatment.
4. Sampling schedules are restricted to standard of care visits amongst a patient cohort treated for drug-sensitive pulmonary TB.
5. Findings will need validation using data from patients with different forms of disease and in other geographical regions.

Keywords: Tuberculosis, drug-sensitive tuberculosis, pharmacokinetics, pharmacokinetics-pharmacodynamics, microbiology, transcriptomics, isoniazid, rifampicin, pyrazinamide, ethambutol.

Word count: 1878

For peer review only

Introduction

Globally in 2019, an estimated 10.0 (9.0-11.0) million people fell ill with tuberculosis (TB). In the same year approximately 1.2 (range: 1.1-1.3) million and 208,000 (range: 177,000-242,000) HIV negative and positive people died from TB. [1] The treatment of TB comprises a combination of antibiotics to optimise the effect and mitigate the emergence of resistance. It currently takes six months minimum to treat drug sensitive TB (DS-TB) or multi drug resistant TB (MDR-TB). [2,3] However, it has been shown that for 80% of patients a shorter treatment duration suffices [4] although there are currently no biomarkers that can accurately identify these individuals. Such a biomarker could for example help to identify patients, within the first two months of treatment, that need to continue the intensive phase as they are not responding well. It could also help improve the design of prospective clinical trials investigating rationally-designed effective, shorter and/or individualised treatment options.

Several approaches, including measuring early bactericidal activity or host immune response, to predict favourable outcomes have been tested. [5–7] Previous research shows that steeper bacterial kill-curves and bacterial kill-curves that are steep for longer correlate with microbiological cure at the end of treatment. Re-occurrence (i.e. re-infection and relapse) was associated with less steep bacterial kill-curves. [8] These findings are consistent with work by others, which demonstrated that detectable *Mycobacterium tuberculosis* (*Mtb*) in sputum two and three months into treatment, i.e. culture conversion, as a predictor for treatment outcome is not good enough for trial-level outcome predictions. [5] The predictive value of culture conversion is even less helpful for individual patients. [6]

Host immune response markers such as seen in the whole blood transcriptome can discriminate between active TB disease and healthy controls. [9] As the expression of certain gene signatures resolves during treatment of active TB [10,11], this information could form the basis for identifying a biomarker of early response to treatment. Although has been reported in patients with drug-sensitive TB; early predictions (e.g., during week 1 and week 4 of treatment) of microbiological cure at the end of treatment were not accurate enough for clinical or trial application. [7]

For peer review only

Interactions between the longitudinal markers e.g., antibiotic exposure, sputum bacillary load and host blood transcriptome within the same patient have to date not been evaluated, even though a combination of antibiotic exposure, sputum bacillary load and host immune response would be expected to follow a composite pattern. The aim of this study is to explore a composite bio-marker for response to treatment of TB. The primary objective is to investigate correlations between antibiotic exposure and sputum bacillary load and host immune response; with a secondary objective to explore trends between the aforementioned correlations and treatment outcome.

Methods and analysis

This is a prospective, longitudinal and repeated-measure study that plans to recruit 210 drug sensitive pulmonary TB patients in London, United Kingdom. Patients will be followed during the 6 month treatment period at pre-defined visits. Formal follow-up visits are not usually scheduled during a 12 month post-treatment period, and instead patients are encouraged to contact the London TB Services immediately when signs and symptoms of possible TB disease re-appear. Patients will be linked to the national TB database to enable monitoring for relapse or reinfection (Table 1).

Table1: Study design.

D: day, W: week and M: month	D0	D14	W4	W8	W12	M4	M5	M6	Follow-up[a]
Consent	✓								
Medical history	✓								
Physical exam	✓								
Body weight measure		✓	✓	✓	✓	✓	✓	✓	✓
Laboratory test[b]	✓	✓	✓	✓	✓	✓	✓	✓	✓
Pregnancy test	✓								
Sputum sampling[c]	✓	✓	✓	✓	✓	✓	✓	✓	✓
Blood plasma sampling		✓		✓				✓	
Whole blood sampling	✓	✓		✓				✓	✓[d]

For peer review only

Medication record box[e]	✓

[a] Passive follow-up phase, patients will be linked to the national TB database to enable monitoring of relapse or reinfection, and are asked to contact the London TB Services when signs and symptoms of possible TB disease re-appear. [b] Including full blood count, liver function tests (hepatic transaminases and total bilirubin), bio-chemistry (urea and electrolytes), glucose. [c] To be conducted until two consecutive negative samples; and sampling may be resumed when there are signs of treatment failure or relapse. [d] To be performed only in case of a recurrence, i.e. a positive sputum smear or culture after patient being successfully treated at month 6 (M6). [e] Medication record box, explain how to use it at the first visit.

Study site selection

TB notification numbers and rates display a steady decline in England since 2011. The majority of TB cases are reported in London (2018: n=1,691 and rate=19.0/100,000) and other major cities such as Birmingham (West Midlands 2018: n=613 and rate=10.4/100,000). Co-infection with HIV is rare amongst TB patients in England at 2.7% in 2018. Isoniazid and Multi Drug Resistant(MDR)/Rifampicin Resistant (RR) TB are also uncommon in England at 8% and 1.6% in 2018. Despite the most recent numbers being low in England, the prevalence of TB in London remains high compared to other major Western European cities. [12]

The relatively high prevalence of TB in London, in combination with well resourced TB clinics, enables us to conduct this observational cohort. Serial sputum bacillary load quantification is common as part of standard of care, whilst drug level measurements are now more frequent given their value in determining if therapeutic drug levels have been achieved [13]. A limited amount of additional measures are therefore needed to enable us to answer the proposed research questions. These include: whole blood sampling for host blood transcriptomics and precise adherence monitoring using medication record boxes.

The London TB Services is organised into five geographical groupings. We intend to recruit from all London sectors. This will provide a diverse study population.

For peer review only

For peer review only - http://bmjopen.bmj.com/site/about/guidelines.xhtml

Participant eligibility criteria and recruitment

Individuals attending the study clinics that meet all inclusion and none of the exclusion criteria are given patient information sheets. If the patient is willing to take part, then written consent will be obtained.

- **Inclusion criteria**
  - Male or female $\geq$ 18 years
  - Sputum sample suggesting *M. tuberculosis* based on culture or molecular test
  - Starting a pulmonary isoniazid and rifampicin susceptible *M. tuberculosis* treatment
  - Permission to be linked to PHE national TB database
  - Willingness to attend study visits
  - Completed written consent
- **Exclusion criteria**
  - Pregnant or breast-feeding
  - Social/medical conditions making study participation unsafe
  - Not eligible to start a course of drug sensitive TB treatment

Assessment of objectives

In order to study both primary and secondary objectives this study collects, in addition to clinical data obtained as part of standard of care, information on adherence to treatment and host blood transcriptomic analysis.

Sample size assumptions

This is a feasibility study, and given the primary objective, the sample size was determined to power accurate and precise estimation of nonlinear mixed-effects model parameters that describe longitudinal pharmacological, microbiological and immunological data. At a sample size of 210

For peer review only

there is power to detect a correlation ≥ 0.19 between the different markers with 80% power and a 0.05 type 1 error rate.

The secondary objective aims to separate composite biomarkers between patients that had or had not reached microbiological cure at the end of treatment, or had or had not finished treatment at six months, and patients that had or had no relapse during the first year after successfully finishing treatment. Assuming a 4.8% failure rate, defined as still on treatment after 12 months [12], accounting for 10% drop out [12], the study design is powered to detect a difference of 0.96 standard deviations in composite markers with 189 patients at 80% power with an 0.05 type 1 error rate.

Clinical covariates

Clinical covariates that are routinely collected as part of standard care will be collected from clinical notes and stored in a REDCap database. [14,15] These covariates include: a medical history check, physical exam and pregnancy test at the D0 visit and at all consecutive visits bodyweight and laboratory tests including full blood count, liver function tests (hepatic transaminases and total bilirubin), urea and electrolytes and glucose (Table 1).

Evaluation of adherence

Patients taking part in this study will be given a medication record box to monitor the date and time that drugs have been taken. [16] Patients will be reminded by phone call or text about the clinic appointment the day before, if they have previously forgotten to bring their medication record boxes with them.

Antibiotic plasma concentrations

Whole blood (0.5-1.0 mL) will be collected at ~2 hours post drug intake on the D14, M2 and M6 visit (Table 1). Plasma will be separated by centrifugation (at 1,500 g for 10 minutes). Samples will subsequently be stored at -70°C, within 24 hours after collection, until shipment on dry ice for analysis.

For peer review only

Antibiotic plasma concentrations will be quantified using a LC-MS/MS as per standard of care protocols.

Sputum bacillary load

In line with usual practice patients will be asked to produce 2-3 sputum samples (2.5 mL) over a 24 hour period at the D0, D14 and M1 visit and then monthly until two negative consecutive sputum samples have been obtained. Where possible sputum will be induced if patients stop coughing prior to two negative consecutive sputum samples have been obtained. Sputum bacterial load will be quantified using MGIT Time To Positivity within 48 hours of production.

Isolates at base line and subsequent treatment failure isolates will be subject to Whole Genome Sequence analysis.

Host blood gene signature expressions

Whole blood (3 mL) will be collected directly into Tempus Blood RNA Tubes from patients at the D0, D14, M2 and M6 visit for host blood transcriptomics. Samples will be stored in the freezer (-20°C) until analysis.

Expression of validated gene signatures, comprising 35 genes, will be quantified using NanoString ® [17,18].

Modelling and statistics

A detailed analysis plan will be developed, but in brief nonlinear mixed-effects models will be used for the primary objective. The first component comprises pharmacokinetic models that characterise how the drugs are absorbed in, distributed through, metabolised and eliminated from the body. [8] The second component will comprise a pharmacodynamic model that characterises clearance of bacillary load from sputum by the rate of elimination and the loss of steepness in bacillary killing over the course of treatment, i.e. mono- or bi-phasic decay. [8] The third component comprises nonlinear mixed-effects models that characterise resolution of gene expression for selected

For peer review only

For peer review only - http://bmjopen.bmj.com/site/about/guidelines.xhtml

signatures during the course of treatment, which may be mono- or bi-phasic. [9,17,18] All three model components consist of a structural model component and variability component that separates random variability between patients from residual variability. With these three types of models and a covariate modelling approach, drug levels-bacterial load correlations and drug levels-host immune response associations will be characterised. [19,20] Partial time series data, e.g. patients switching treatment due to development of adverse events, will be retained for analysis.

A multivariate generalised logistic regression model will be used for the secondary objective, to explore a composite biomarker, comprising the aforementioned matrices, that best predicts microbiological cure at the end of treatment. The candidate composite biomarker's power to predict relapse during the 12 months following successful treatment will also be explored using a Cox Proportional Hazard model.

All aforementioned analyses will be performed using k-fold validation with the training partition of a proposed train:test split of 70:30 to obtain a more reliable predictive performance of the algorithm on the test split.[21] Furthermore, data from this study will need to be combined with that from other studies to further study the generalisability of the findings.

Project status

The Royal Free London NHS Foundation Trust will be the first site to recruit patients with subsequent roll-out to other London sites.

Ethics and dissemination

This observational cohort study has been approved by the UK's Health Research Authority Research Ethics Committee (reference: 20/SW/0007).

Results will be disseminated through engagement with institutes/companies developing novel anti-TB treatment combinations and via presentation at scientific conferences, as well as through publication in open-access peer reviewed journals.

For peer review only

Author contributions

FK and ML conceived the study and designed the protocol together with IA, HE, TMcH and MN. FK wrote and all other authors critically reviewed the manuscript.

Data statement

A technical appendix, statistical code, and an anonymised dataset will be made available.

Patient and public involvement

Patients or the public were not involved in the design, or conduct, or reporting, or dissemination plans of the research.

Funding statement

The study is funded through a Sir Henry Dale Fellowship jointly funded by the Wellcome Trust and the Royal Society (Grant Number 220587/Z/20/Z) and part of the medication record boxes were funded through a UKRI Medical Research Council fellowship (Grant Number MR/P014534/1), both awarded to FK.

Competing interests

None to declare.

References

1 Organization WH. *Global tuberculosis report 2020*. World Health Organization 2020.

2 Organization WH, Health Organization) STI (World. *Treatment of tuberculosis: Guidelines*. World Health Organization 2010.

3 Organization WH, others. *WHO consolidated guidelines on drug-resistant tuberculosis treatment*. World Health Organization 2019.

For peer review only

4 Fox W, Ellard GA, Mitchison DA. Studies on the treatment of tuberculosis undertaken by the british medical research council tuberculosis units, 1946–1986, with relevant subsequent publications. *The International Journal of Tuberculosis and Lung Disease* 1999;**3**:S231–79.

5 Phillips PP, Fielding K, Nunn AJ. An evaluation of culture results during treatment for tuberculosis as surrogate endpoints for treatment failure and relapse. *PloS one* 2013;**8**:e63840.

6 Phillips PP, Mendel CM, Burger DA *et al.* Limited role of culture conversion for decision-making in individual patient care and for advancing novel regimens to confirmatory clinical trials. *BMC medicine* 2016;**14**:19.

7 Thompson EG, Du Y, Malherbe ST *et al.* Host blood rna signatures predict the outcome of tuberculosis treatment. *Tuberculosis* 2017;**107**:48–58.

8 Kloprogge F, Mwandumba HC, Banda G *et al.* Longitudinal pharmacokinetic-pharmacodynamic biomarkers correlate with treatment outcome in drug-sensitive pulmonary tuberculosis: A population pharmacokinetic-pharmacodynamic analysis. In: *Open forum infectious diseases*. Oxford University Press US 2020. ofaa218.

9 Roe JK, Thomas N, Gil E *et al.* Blood transcriptomic diagnosis of pulmonary and extrapulmonary tuberculosis. *JCI insight* 2016;**1**:e87238.

10 Bloom CI, Graham CM, Berry MP *et al.* Detectable changes in the blood transcriptome are present after two weeks of antituberculosis therapy. *PloS one* 2012;**7**:e46191.

11 Cliff JM, Lee J-S, Constantinou N *et al.* Distinct phases of blood gene expression pattern through tuberculosis treatment reflect modulation of the humoral immune response. *The Journal of infectious diseases* 2013;**207**:18–29.

12 England PH. *Tuberculosis in england: 2020 report*. Public Health England 2020.

For peer review only

13 Nahid P, Dorman SE, Alipanah N *et al.* Official american thoracic society/centers for disease control and prevention/infectious diseases society of america clinical practice guidelines: Treatment of drug-susceptible tuberculosis. *Clinical Infectious Diseases* 2016;**63**:e147–95.

14 Harris PA, Taylor R, Thielke R *et al.* Research electronic data capture (redcap)—a metadata-driven methodology and workflow process for providing translational research informatics support. *Journal of biomedical informatics* 2009;**42**:377–81.

15 Harris PA, Taylor R, Minor BL *et al.* The redcap consortium: Building an international community of software platform partners. *Journal of biomedical informatics* 2019;**95**:103208.

16 Subbaraman R, Mondesert L de, Musiimenta A *et al.* Digital adherence technologies for the management of tuberculosis therapy: Mapping the landscape and research priorities. *BMJ global health* 2018;**3**.

17 Gupta RK, Turner CT, Venturini C *et al.* Concise whole blood transcriptional signatures for incipient tuberculosis: A systematic review and patient-level pooled meta-analysis. *The Lancet Respiratory Medicine* 2020;**8**:395–406.

18 Turner CT, Gupta RK, Tsaliki E *et al.* Blood transcriptional biomarkers for active pulmonary tuberculosis in a high-burden setting: A prospective, observational, diagnostic accuracy study. *The Lancet Respiratory Medicine* 2020;**8**:407–19.

19 Nielsen EI, Friberg LE. Pharmacokinetic-pharmacodynamic modeling of antibacterial drugs. *Pharmacological reviews* 2013;**65**:1053–90.

20 Upton R, Mould D. Basic concepts in population modeling, simulation, and model-based drug development: Part 3—introduction to pharmacodynamic modeling methods. *CPT: pharmacometrics & systems pharmacology* 2014;**3**:1–16.

21 Bengio Y, Grandvalet Y. No unbiased estimator of the variance of k-fold cross-validation. *Journal of machine learning research* 2004;**5**:1089–105.

For peer review only

BMJ Open

Exploring a combined biomarker for tuberculosis treatment response: protocol for a prospective observational cohort study

Journal:	*BMJ Open*
Manuscript ID	bmjopen-2021-052885.R1
Article Type:	Protocol
Date Submitted by the Author:	17-Jun-2021
Complete List of Authors:	Kloprogge, Frank; University College London<br>Abubakar, Ibrahim; Health Protection Agency, Tuberculosis Section, Centre for Infections<br>Esmail, Hanif; University College London,<br>Hack, Vanessa ; University College London, Institute for Global Health<br>Kunst, Heinke; Queen Mary University of London, Department of Respiratory Medicine<br>McHugh, Timothy; Centre for Clinical Microbiology, Research Department of Infection, Royal Free Campus, University College London<br>Noursadeghi, Mahdad; University College London<br>Surey, Julian; University College London<br>Tiberi, Simon; Barts Health NHS Trust, Blizard Institute, Queen Mary University of London, Department of Infection<br>Lipman, Marc; Royal Free Campus, Respiratory Medicine
**Primary Subject Heading**:	Pharmacology and therapeutics
Secondary Subject Heading:	Infectious diseases
Keywords:	Tuberculosis < INFECTIOUS DISEASES, BACTERIOLOGY, CLINICAL PHARMACOLOGY

SCHOLARONE™ Manuscripts

I, the Submitting Author has the right to grant and does grant on behalf of all authors of the Work (as defined in the below author licence), an exclusive licence and/or a non-exclusive licence for contributions from authors who are: i) UK Crown employees; ii) where BMJ has agreed a CC-BY licence shall apply, and/or iii) in accordance with the terms applicable for US Federal Government officers or employees acting as part of their official duties; on a worldwide, perpetual, irrevocable, royalty-free basis to BMJ Publishing Group Ltd ("BMJ") its licensees and where the relevant Journal is co-owned by BMJ to the co-owners of the Journal, to publish the Work in this journal and any other BMJ products and to exploit all rights, as set out in our licence.

The Submitting Author accepts and understands that any supply made under these terms is made by BMJ to the Submitting Author unless you are acting as an employee on behalf of your employer or a postgraduate student of an affiliated institution which is paying any applicable article publishing charge ("APC") for Open Access articles. Where the Submitting Author wishes to make the Work available on an Open Access basis (and intends to pay the relevant APC), the terms of reuse of such Open Access shall be governed by a Creative Commons licence – details of these licences and which Creative Commons licence will apply to this Work are set out in our licence referred to above.

Other than as permitted in any relevant BMJ Author's Self Archiving Policies, I confirm this Work has not been accepted for publication elsewhere, is not being considered for publication elsewhere and does not duplicate material already published. I confirm all authors consent to publication of this Work and authorise the granting of this licence.

For peer review only

Exploring a combined biomarker for tuberculosis treatment response: protocol for a prospective observational cohort study

Frank Kloprogge (1), Ibrahim Abubakar (1), Hanif Esmail (1,2), Vanessa Hack (1), Heinke Kunst (3), Timothy D. McHugh (4), Mahdad Noursadeghi (5), Julian Surey (1), Simon Tiberi (3,6), Marc Lipman (7,8)

1 Institute for Global Health, University College London, United Kingdom.

2 Medical Research Council Clinical Trials Unit, University College London, London, United Kingdom.

3 Blizard Institute, Barts and The London School of Medicine and Dentistry, Queen Mary University of London, UK

4 UCL Centre for Clinical Microbiology, Division of Infection & Immunity, University College London, London, United Kingdom.

5 Division of Infection and Immunity, University College London, London, United Kingdom.

6 Division of Infection, Royal London Hospital, Barts Health NHS Trust, London, United Kingdom.

7 Royal Free London NHS Foundation Trust, London, United Kingdom.

8 UCL Respiratory, Division of Medicine, University College London, London, United Kingdom.

Corresponding author:

Frank Kloprogge

30 Guilford Street

London

WC1N 1EH

United Kingdom

Tel: +44 (0)20 7905 2889

Email: f.kloprogge@ucl.ac.uk

For peer review only

Abstract

Introduction

An improved understanding of factors explaining tuberculosis (TB) treatment response is urgently needed to help clinicians optimise and personalise treatment and assist scientists undertaking novel treatment regimen trials. Promising outcome proxy measures, including sputum bacillary load and host immune response, are widely reported with variable results. However, they have not been studied together in combination with antibiotic exposure. The aim of this observational cohort study is to investigate which antibiotic exposures correlate with sputum bacillary load and which with the host immune response. Subsequently, we will explore if these correlations can be used to inform a candidate combined biomarker predicting cure.

Methods and analysis

All patients aged ≥ 18, diagnosed with drug sensitive pulmonary TB (culture or molecular test), eligible for standard anti-tuberculosis treatment, at selected London, UK TB Services will be invited to participate in this observational cohort study (target sample size = 210). Patients will be asked to give blood for host transcriptomics and antibiotic plasma exposure, in addition to standard of care sputum samples for bacillary load. Antibiotic plasma concentrations will be quantified using a validated LC-MS/MS assay, and sputum bacillary load by Mycobacterial Growth Incubator Tube (MGIT) time to positivity (TTP). Expression from a total of 35 prespecified host blood genes will be quantified using NanoString®. Antibiotic exposure, sputum bacillary load and host blood transcriptomic time series data will be analysed using nonlinear mixed-effects models. Correlations between combinations of longitudinal biomarkers and microbiological cure at the end of treatment and remaining relapse-free for one year thereafter will be analysed using logistic regression or Cox proportional hazard models.

For peer review only - http://bmjopen.bmj.com/site/about/guidelines.xhtml

48 ***Ethics and dissemination***

49 The observational cohort study has been approved by the UK's HRA REC (20/SW/0007). Written
50 informed consent will be obtained. Results will be disseminated via publication, presentation and
51 through engagement with institutes/companies developing novel anti-TB treatment combinations.

52

For peer review only

Article summary

1. This study explores for the first time correlations between antibiotic exposure and both sputum tuberculosis (TB) bacillary load and host immune response.
2. This enables correlations between different combinations of longitudinal markers of TB treatment response and TB treatment outcome to be explored.
3. Such correlations can form the basis for a candidate biomarker that predicts cure from TB during the intensive phase of treatment.
4. Sampling schedules are restricted to standard of care visits amongst a patient cohort treated for drug-sensitive pulmonary TB.
5. Findings will need validation using data from patients with different forms of disease and in other geographical regions.

Keywords: Tuberculosis, drug-sensitive tuberculosis, pharmacokinetics, pharmacokinetics-pharmacodynamics, microbiology, transcriptomics, isoniazid, rifampicin, pyrazinamide, ethambutol.

Word count: 2085

For peer review only

Introduction

Globally in 2019, an estimated 10.0 (9.0-11.0) million people fell ill with tuberculosis (TB). In the same year approximately 1.2 (range: 1.1-1.3) million and 208,000 (range: 177,000-242,000) HIV negative and positive people died from TB. [1] The treatment of TB comprises a combination of antibiotics to optimise the effect and mitigate the emergence of resistance. It currently takes six months minimum to treat drug sensitive TB (DS-TB) or multi drug resistant TB (MDR-TB). [2,3] However, it has been shown that for 80% of patients a shorter treatment duration suffices [4] although there are currently no biomarkers that can accurately identify these individuals. Such a biomarker could for example help to identify patients, within the first two months of treatment, that need to continue the intensive phase as they are not responding well. It could also help improve the design of prospective clinical trials investigating rationally-designed effective, shorter and/or individualised treatment options.

Several approaches, including measuring early bactericidal activity or host immune response, to predict favourable outcomes have been tested. [5–7] Previous research shows that steeper bacterial kill-curves and bacterial kill-curves that are steep for longer correlate with microbiological cure at the end of treatment. Re-occurrence (i.e. re-infection and relapse) was associated with less steep bacterial kill-curves. [8] These findings are consistent with work by others, which demonstrated that detectable *Mycobacterium tuberculosis* (*Mtb*) in sputum two and three months into treatment, i.e. culture conversion, as a predictor for treatment outcome is not good enough for trial-level outcome predictions. [5] The predictive value of culture conversion is even less helpful for individual patients. [6]

Host immune response markers such as seen in the whole blood transcriptome can discriminate between active TB disease and healthy controls. [9] As the expression of certain gene signatures resolves during treatment of active TB [10,11], this information could form the basis for identifying a biomarker of early response to treatment. Although has been reported in patients with drug-sensitive TB; early predictions (e.g., during week 1 and week 4 of treatment) of microbiological cure at the end of treatment were not accurate enough for clinical or trial application. [7]

For peer review only

Interactions between the longitudinal markers e.g., antibiotic exposure, sputum bacillary load and host blood transcriptome within the same patient have to date not been evaluated, even though a combination of antibiotic exposure, sputum bacillary load and host immune response would be expected to follow a composite pattern. The aim of this study is to explore a composite bio-marker for response to treatment of TB. The primary objective is to investigate correlations between antibiotic exposure and sputum bacillary load and host immune response; with a secondary objective to explore trends between the aforementioned correlations and treatment outcome.

Methods and analysis

This is a prospective, longitudinal and repeated-measure study that plans to recruit 210 drug sensitive pulmonary TB patients in London, United Kingdom. Patients will be followed during the 6 month treatment period at pre-defined visits. Formal follow-up visits are not usually scheduled during a 12 month post-treatment period, and instead patients are encouraged to contact the London TB Services immediately when signs and symptoms of possible TB disease re-appear. Patients will be linked to the national TB database to enable monitoring for relapse or reinfection (Table 1).

Table1: Study design.

D: day, W: week and M: month	D0	D14	W4	W8	W12	M4	M5	M6	Follow-up[a]
Consent	✓								
Medical history	✓								
Physical exam	✓								
Body weight measure		✓	✓	✓	✓	✓	✓	✓	✓
Laboratory test[b]	✓	✓	✓	✓	✓	✓	✓	✓	✓
Pregnancy test	✓								
Sputum sampling[c]	✓	✓	✓	✓	✓	✓	✓	✓	✓
Blood plasma sampling		✓		✓				✓	
Whole blood sampling	✓	✓		✓				✓	✓[d]

For peer review only

For peer review only - http://bmjopen.bmj.com/site/about/guidelines.xhtml

Medication record box[e]	✓

[a] Passive follow-up phase, patients will be linked to the national TB database to enable monitoring of relapse or reinfection, and are asked to contact the London TB Services when signs and symptoms of possible TB disease re-appear. [b] Including full blood count, liver function tests (hepatic transaminases and total bilirubin), bio-chemistry (urea and electrolytes), glucose. [c] To be conducted until two consecutive negative samples; and sampling may be resumed when there are signs of treatment failure or relapse. [d] To be performed only in case of a recurrence, i.e. a positive sputum smear or culture after patient being successfully treated at month 6 (M6). [e] Medication record box, explain how to use it at the first visit.

Study site selection

TB notification numbers and rates display a steady decline in England since 2011. The majority of TB cases are reported in London (2018: n=1,691 and rate=19.0/100,000) and other major cities such as Birmingham (West Midlands 2018: n=613 and rate=10.4/100,000). Co-infection with HIV is rare amongst TB patients in England at 2.7% in 2018. Isoniazid and Multi Drug Resistant(MDR)/Rifampicin Resistant (RR) TB are also uncommon in England at 8% and 1.6% in 2018. Despite the most recent numbers being low in England, the prevalence of TB in London remains high compared to other major Western European cities. [12]

The relatively high prevalence of TB in London, in combination with well resourced TB clinics, enables us to conduct this observational cohort. Serial sputum bacillary load quantification is common as part of standard of care, whilst drug level measurements are now more frequent given their value in determining if therapeutic drug levels have been achieved [13]. A limited amount of additional measures are therefore needed to enable us to answer the proposed research questions. These include: whole blood sampling for host blood transcriptomics and precise adherence monitoring using medication record boxes.

For peer review only

For peer review only - http://bmjopen.bmj.com/site/about/guidelines.xhtml

The London TB Services is organised into five geographical groupings. We intend to recruit from all London sectors. This will provide a diverse study population. The study started on February 23 2021 and we expect to reach our target sample size in 24 months.

Participant eligibility criteria and recruitment

Individuals attending the study clinics that meet all inclusion and none of the exclusion criteria are given patient information sheets. If the patient is willing to take part, then written consent will be obtained.

- **Inclusion criteria**
  - Male or female $\geq$ 18 years
  - Sputum sample suggesting *M. tuberculosis* based on culture or molecular test
  - Starting a pulmonary isoniazid and rifampicin susceptible *M. tuberculosis* treatment
  - Permission to be linked to PHE national TB database
  - Willingness to attend study visits
  - Completed written consent
- **Exclusion criteria**
  - Pregnant or breast-feeding
  - Social/medical conditions making study participation unsafe
  - Not eligible to start a course of drug sensitive TB treatment

Drug sensitivity testing at baseline is part of NHS standard care. This enables only patients with isoniazid and rifampicin susceptible *M. tuberculosis* infections to be enrolled.

Assessment of objectives

In order to study both primary and secondary objectives this study collects, in addition to clinical data obtained as part of standard of care, information on adherence to treatment and host blood transcriptomic analysis.

For peer review only

Sample size assumptions

Given the primary objective, the sample size for this study was determined to power accurate and precise estimation of nonlinear mixed-effects model parameters that describe longitudinal pharmacological, microbiological and immunological data. At a sample size of 210 there is power to detect a correlation $\geq$ 0.19 between the different markers with 80% power and a 0.05 type 1 error rate.

The secondary objective aims to separate composite biomarkers between patients that had or had not reached microbiological cure at the end of treatment, or had or had not finished treatment at six months, and patients that had or had no relapse during the first year after successfully finishing treatment. Assuming a 4.8% failure rate, defined as still on treatment after 12 months [12], accounting for 10% drop out [12], the study design is powered to detect a difference of 0.96 standard deviations in composite markers with 189 patients at 80% power with an 0.05 type 1 error rate.

Clinical covariates

Clinical covariates that are routinely collected as part of standard care will be collected from clinical notes and stored in a REDCap database. [14,15] These covariates include: a medical history check, physical exam and pregnancy test at the D0 visit and at all consecutive visits bodyweight and laboratory tests including full blood count, liver function tests (hepatic transaminases and total bilirubin), urea and electrolytes and glucose (Table 1).

Evaluation of adherence

Patients taking part in this study will be given a medication record box to monitor the date and time that drugs have been taken. [16] Patients will be reminded by phone call or text about the clinic appointment the day before, if they have previously forgotten to bring their medication record boxes with them. This will be alongside standard NHS care, where patients who on initial or subsequent risk assessment are felt to be at high risk of poor adherence will be offered support

For peer review only

182 with a range of options including Directly Observed Therapy, Video Observed Therapy and dosette
183 boxes.

184 *Antibiotic plasma concentrations*

185 Whole blood (0.5-1.0 mL) will be collected at ~2 hours post drug intake on the D14, M2 and M6
186 visit (Table 1). Plasma will be separated by centrifugation (at 1,500 g for 10 minutes). Samples will
187 subsequently be stored at -70°C, within 24 hours after collection, until shipment on dry ice for
188 analysis.

189 Antibiotic plasma concentrations will be quantified using a LC-MS/MS as per standard of care
190 protocols.

191 *Sputum bacillary load*

192 In line with usual practice patients will be asked to produce 2-3 sputum samples (2.5 mL) over a 24
193 hour period at the D0, D14 and M1 visit and then monthly until two negative consecutive sputum
194 samples have been obtained. Where possible sputum will be induced if patients stop coughing
195 prior to two negative consecutive sputum samples have been obtained. Sputum bacterial load will
196 be quantified using MGIT Time To Positivity within 48 hours of production.

197 Isolates at base line and subsequent treatment failure isolates will be subject to Whole Genome
198 Sequence analysis.

199 *Host blood gene signature expressions*

200 Whole blood (3 mL) will be collected directly into Tempus Blood RNA Tubes from patients at the
201 D0, D14, M2 and M6 visit for host blood transcriptomics. Samples will be stored in the freezer (-
202 20°C) until analysis.

203 Expression of validated gene signatures, comprising 35 genes, will be quantified using NanoString
204 ® [17,18].

For peer review only

Modelling and statistics

A detailed analysis plan will be developed, but in brief nonlinear mixed-effects models will be used for the primary objective. The first component comprises pharmacokinetic models that characterise how the drugs are absorbed in, distributed through, metabolised and eliminated from the body. [8] The second component will comprise a pharmacodynamic model that characterises clearance of bacillary load from sputum by the rate of elimination and the loss of steepness in bacillary killing over the course of treatment, i.e. mono- or bi-phasic decay. [8] The third component comprises nonlinear mixed-effects models that characterise resolution of gene expression for selected signatures during the course of treatment, which may be mono- or bi-phasic. [9,17,18] All three model components consist of a structural model component and variability component that separates random variability between patients from residual variability. With these three types of models and a covariate modelling approach, drug levels-bacterial load correlations and drug levels-host immune response associations will be characterised. [19,20] Partial time series data, e.g. patients switching treatment due to development of adverse events, will be retained for analysis.

A multivariate generalised logistic regression model will be used for the secondary objective, to explore a composite biomarker, comprising the aforementioned matrices, that best predicts microbiological cure at the end of treatment. The candidate composite biomarker's power to predict relapse during the 12 months following successful treatment will also be explored using a Cox Proportional Hazard model.

All aforementioned analyses will be performed using k-fold validation with the training partition of a proposed train:test split of 70:30 to obtain a more reliable predictive performance of the algorithm on the test split.[21] Furthermore, data from this study will need to be combined with that from other studies to further study the generalisability of the findings.

Patient and Public Involvement

No patient and public involved.

For peer review only

Project status

The Royal Free London NHS Foundation Trust will be the first site to recruit patients with subsequent roll-out to other London sites.

Ethics and dissemination

This observational cohort study has been approved by the UK's Health Research Authority Research Ethics Committee (reference: 20/SW/0007). Written informed consent will be obtained from study participants.

Results will be disseminated through engagement with institutes/companies developing novel anti-TB treatment combinations and via presentation at scientific conferences, as well as through publication in open-access peer reviewed journals.

Author contributions

FK and ML conceived the study and designed the protocol together with IA, HE, TMcH and MN. FK wrote and IA, HE, VH, HK, TMcH, MN, JS, ST and ML critically reviewed the manuscript.

Data statement

A technical appendix with statistical code will be made available with publication and the anonymised dataset will be made available upon reasonable request.

Funding statement

The study is funded through a Sir Henry Dale Fellowship jointly funded by the Wellcome Trust and the Royal Society (Grant Number 220587/Z/20/Z) and part of the medication record boxes were funded through a UKRI Medical Research Council fellowship (Grant Number MR/P014534/1), both awarded to FK.

For peer review only

Competing interests

None to declare.

References

1 *Global tuberculosis report 2020*. World Health Organization 2020.

2 *Treatment of tuberculosis: guidelines*. World Health Organization 2010.

3 *WHO consolidated guidelines on drug-resistant tuberculosis treatment*. World Health Organization 2019.

4 Fox W, Ellard GA, Mitchison DA. Studies on the treatment of tuberculosis undertaken by the british medical research council tuberculosis units, 1946–1986, with relevant subsequent publications. *The International Journal of Tuberculosis and Lung Disease* 1999;**3**:S231–79.

5 Phillips PP, Fielding K, Nunn AJ. An evaluation of culture results during treatment for tuberculosis as surrogate endpoints for treatment failure and relapse. *PloS one* 2013;**8**:e63840.

6 Phillips PP, Mendel CM, Burger DA, *et al.* Limited role of culture conversion for decision-making in individual patient care and for advancing novel regimens to confirmatory clinical trials. *BMC medicine* 2016;**14**:19.

7 Thompson EG, Du Y, Malherbe ST, *et al.* Host blood RNA signatures predict the outcome of tuberculosis treatment. *Tuberculosis* 2017;**107**:48–58.

8 Kloprogge F, Mwandumba HC, Banda G, *et al.* Longitudinal pharmacokinetic-pharmacodynamic biomarkers correlate with treatment outcome in drug-sensitive pulmonary tuberculosis: A population pharmacokinetic-pharmacodynamic analysis. In: *Open forum infectious diseases*. Oxford University Press US 2020. ofaa218.

9 Roe JK, Thomas N, Gil E, *et al.* Blood transcriptomic diagnosis of pulmonary and extrapulmonary tuberculosis. *JCI insight* 2016;**1**:e87238.

For peer review only

10 Bloom CI, Graham CM, Berry MP, *et al.* Detectable changes in the blood transcriptome are present after two weeks of antituberculosis therapy. *PloS one* 2012;**7**:e46191.

11 Cliff JM, Lee J-S, Constantinou N, *et al.* Distinct phases of blood gene expression pattern through tuberculosis treatment reflect modulation of the humoral immune response. *The Journal of infectious diseases* 2013;**207**:18–29.

12 England PH. *Tuberculosis in england: 2020 report*. Public Health England 2020.

13 Nahid P, Dorman SE, Alipanah N, *et al.* Official american thoracic society/centers for disease control and prevention/infectious diseases society of america clinical practice guidelines: Treatment of drug-susceptible tuberculosis. *Clinical Infectious Diseases* 2016;**63**:e147–95.

14 Harris PA, Taylor R, Thielke R, *et al.* Research electronic data capture (REDCap)—a metadata-driven methodology and workflow process for providing translational research informatics support. *Journal of biomedical informatics* 2009;**42**:377–81.

15 Harris PA, Taylor R, Minor BL, *et al.* The REDCap consortium: Building an international community of software platform partners. *Journal of biomedical informatics* 2019;**95**:103208.

16 Subbaraman R, Mondesert L de, Musiimenta A, *et al.* Digital adherence technologies for the management of tuberculosis therapy: Mapping the landscape and research priorities. *BMJ global health* 2018;**3**.

17 Gupta RK, Turner CT, Venturini C, *et al.* Concise whole blood transcriptional signatures for incipient tuberculosis: A systematic review and patient-level pooled meta-analysis. *The Lancet Respiratory Medicine* 2020;**8**:395–406.

18 Turner CT, Gupta RK, Tsaliki E, *et al.* Blood transcriptional biomarkers for active pulmonary tuberculosis in a high-burden setting: A prospective, observational, diagnostic accuracy study. *The Lancet Respiratory Medicine* 2020;**8**:407–19.

For peer review only

19 Nielsen EI, Friberg LE. Pharmacokinetic-pharmacodynamic modeling of antibacterial drugs. *Pharmacological reviews* 2013;**65**:1053–90.

20 Upton R, Mould D. Basic concepts in population modeling, simulation, and model-based drug development: Part 3—introduction to pharmacodynamic modeling methods. *CPT: pharmacometrics & systems pharmacology* 2014;**3**:1–6.

21 Bengio Y, Grandvalet Y. No unbiased estimator of the variance of k-fold cross-validation. *Journal of machine learning research* 2004;**5**:1089–105.

For peer review only
